# Risk of COVID-19 due to Shortage of Personal Protective Equipment

**DOI:** 10.7759/cureus.8837

**Published:** 2020-06-25

**Authors:** Uday Jain

**Affiliations:** 1 Anesthesiology, San Mateo Medical Center, San Mateo, USA

**Keywords:** covid-19, provider, personal protective equipment, shortage, reprocessing, risk, age, comorbidity, race, infection

## Abstract

The supply of personal protective equipment (PPE) is inadequate throughout the United States and the world. This is especially true of N95 respirators. The cost of PPE is high. There are numerous cases of providers working with inadequate PPE and being disciplined on complaining. In the United States, thousands of providers have contracted COVID-19, in part due to inadequate PPE. Extended use and reuse of N95 respirators has been permitted by the Centers for Disease Control and Prevention (CDC). The N95 respirators can be sterilized utilizing vaporized hydrogen peroxide, ultraviolet germicidal irradiation, or dry heat at 70°C-80°C.

The risk to providers due to inadequate PPE increases with their age and presence of comorbidities. African-Americans and Latinos are at a greater risk. CDC recommends that in the absence of appropriate PPE, “exclude healthcare personnel at higher risk for severe illness from COVID-19 from contact with known or suspected COVID-19 patients.” Providing care without appropriate PPE should not be a condition of employment for any provider, especially for the ones in high-risk category.

## Introduction and background

SARS-CoV-2 is a very infective virus. Providers caring for COVID-19 patients or persons under investigation are at increased risk of contracting the illness. In the United States, thousands of providers have contracted COVID-19. The risk of infection among the providers is greater than the risk among the general population.

The risk increases with age and presence of comorbidities. The risk is greater among African-Americans and Latinos. Appropriate utilization of personal protective equipment (PPE) can mitigate the risk. However, there is a shortage of PPE, especially N95 respirators. Centers for Disease Control and Prevention (CDC) has approved extended use, reuse, and reprocessing of N95 respirators. The providers must ensure that they follow guidelines including handwashing and appropriate use of PPE. 

## Review

Personal protective equipment

For protection against COVID-19, PPE includes head, face, beard, and shoe covers; impervious gown, gloves, respirator, and eye protection. According to Anesthesia Patient Safety Foundation, during airway management, providers should utilize appropriate PPE for all patients [1]. The American Society of Anesthesiologists and other organizations have recommended “all anesthesia professionals should utilize PPE appropriate for aerosol-generating procedures for all patients when working near the airway [2].”

Providers contracting COVID-19

Providers are at a substantial risk for developing COVID-19 and spreading it [3]. According to Morbidity and Mortality Weekly Report (MMWR) of 14th April 2020 by the CDC, 9,282 U.S. providers contracted COVID-19 [4]. Ninety percent of them were not hospitalized. About 0.5% died. Limited data indicated that providers accounted for about 11% of all COVID-19 infections, although they are a much lower percentage of population. According to unpublished accounts, as of 23rd April 2020, more than 21,800 U.S. providers had contracted COVID-19 and 71 had died.

This very high risk causes psychological distress among the providers [5]. It is especially so among younger and more junior workers and those with dependent children [5]. Risk factors for providers contracting COVID-19 include inadequate handwashing, inadequate or improper use of PPE, and prolonged work hours and exposure [6]. Infected providers are vectors for infection. In Wuhan 41% of the COVID-19 cases resulted from hospital-related transmission [7].

Shortage of personal protective equipment

The supply of PPE is inadequate throughout the United States and the rest of the world [8-11]. This is especially true of N95 respirators for providers and even of surgical masks to be utilized by everyone. In some locations, the shortage is critical, forcing the providers to work under unsafe conditions. In part because of the shortage, the cost of PPE is high. Many hospitals are having difficulty paying for PPE.

The U.S. manufacturers are increasing the production of PPE. Attempts are being made to import more PPE. N95 respirators approved by the National Institute for Occupational Safety and Health (NIOSH) but not the U.S. Food and Drug Administration (FDA), and intended for industrial use, are being allowed to be used for medical applications. Activation of the Defense Production Act has allowed the government to direct industry to switch to producing PPE. PPE is also being released from the Strategic National Stockpile which itself is small.

Some of the stockpiled N95 respirators are expired. The elastic band of an expired respirator may not function well. If the band is working, an expired respirator may be usable. Many persons are using home-made surgical masks. The CDC recommends using bandanas if surgical masks are not available [12].

Providers disciplined for complaining

Throughout the United States there are numerous cases of providers working with inadequate PPE and being disciplined on complaining, especially to media [13-15]. According to the New York Times, every major private hospital system and some public hospitals in New York City have sent memos ordering workers not to speak to the media.

There are many disputes between providers and hospitals regarding what is appropriate PPE in each setting. Many hospitals are requiring providers to reuse their own PPE that may no longer be sterile or protective. Some hospitals that do not provide adequate PPE still restrict the providers from using PPE they own and bring to the hospital.

Extended use and reuse of N95 respirators

During shortages, the CDC recommends a maximum period of 8-12 hours of extended use for multiple patients without doffing in between. CDC suggests limiting N95 respirator reuse to no more than five times per device with doffing between uses. A face shield or surgical mask can be utilized to cover the N95 respirator for extended use or reuse.

Reprocessed N95 respirator

During shortages, the respirators can be sterilized [16-17]. Apparatus utilizing vaporized hydrogen peroxide is widely available in hospitals [18-19]. This can allow decontamination of up to four million N95 respirators. After 20 cycles of sterilization, the filtration was not impaired but the mask fit was degraded. Filtration was not impaired after up to 50 cycles of ultraviolet germicidal irradiation but the fit was impaired after an average of three cycles. Dry heat at 70°C-80°C can be used for two cycles of decontamination. 

Alternative solutions

These include securing an anesthesia breathing circuit mask on the face of the provider [20-21]. A HEPA filter can be placed between the mask and the atmosphere. A respirator fashioned from a snorkel mask has received FDA approval. The efficacy of a surgical mask or bandana can be increased by making it fit tightly. This can be achieved by utilizing tape, elastic bands, and other items. More efficient masks may be available in the near future [22-23].

Risk of COVID-19 in the United States

The MMWR dated 31st March 31 2020 reported data on COVID-19 patients in the United States [24]. Among patients aged ≥19 years of age, in 37.6% of the patients, at least one of the following underlying medical conditions was present: diabetes mellitus (10.9%), chronic lung disease (9.2%), cardiovascular disease (9.0%), chronic renal disease, chronic liver disease, immunocompromise, neurologic disease, neurodevelopmental or intellectual disability, pregnancy, smoking, or other chronic disease including cancer and hypertension. The percentage of such patients among all patients not requiring hospitalization was 27%, among all patients requiring hospitalization without ICU admission was 71%, among all patients requiring ICU admission was 78%, and among all dead patients was 94%. Stratification by the number, severity, and level of control of underlying medical conditions is not available.

Table 1 stratifies the risk by age and underlying medical condition. Thus, 22.2% of the patients older than 65 years and with one or more underlying medical conditions were admitted to ICU. Only 2% of the patients younger than 65 years and with no underlying medical condition were admitted to ICU. Older age and an underlying medical condition, each increased the risk about three-fold. Together, they increased the risk by an order of magnitude.

**Table 1:** Percentage of patients stratified by age and presence of underlying medical condition.

Age group (years)	Hospitalized without ICU admission		ICU admission	
	Underlying condition	No underlying condition	Underlying condition	No underlying condition
19-64 years	19.9%	6.7%	9.4%	2.0%
≥65 years	44.5%	18.3%	22.2%	6.3%

Risk of COVID-19 in other countries

A study of patients from China and 37 other countries, published online in Lancet Infect Dis on 30th March 2020, estimated a mortality of 13.4% in patients 80 years and older, 8.6% in patients 70-79 years old, 4% in patients 60-69 years old, 1.25% in those 50-59 years old, and 0.3% in those 40-49 years old [25]. A study of Italian patients estimated a mortality of 20.2% in patients 80 years and older, 12.8% in patients 70-79 years old, 3.5% in patients 60-69 years old, 1% in those 50-59 years old, and 0.4% in those 40-49 years old [26].

Racial differences

Although detailed data are not yet available, African-Americans, Latinos, and other minorities are at a greater risk of contracting COVID-19 [27-28]. If they develop COVID-19, they have a greater risk of mortality. One reason for this is that they have higher rates of comorbidities including hypertension, heart disease, diabetes, and lung disease. According to the U.S. Surgeon General Jerome M. Adams, “the burden of social ills is also contributing.”

Because of financial and other reasons, minorities are likely to get inadequate care and get care later in the disease cycle. Jobs of many African-Americans, Latinos, and other minorities require in-person interaction, precluding teleworking. Many work in service, transportation, and hospitality industries. Many such jobs are considered “essential,” precluding staying home safely. Surgeon General Adams said, “People of color are more likely to live in densely packed areas and in multi-generational housing situations, which create higher risk for spread of highly contagious disease like covid-19.” Many of their homes have health hazards.

Other risk factors

In United States, COVID-19 has infected more women but killed more men [29]. In China, mortality was much higher among men. The cause for this is not elucidated. As the virus can gain entry from a break in the skin, a provider with injury or recent surgery may be at increased risk [30]. Patients with blood group A are at a higher risk of infection and develop more severe symptoms whereas patients with blood type O are at a lower risk [31-32].

Mitigating risk for providers’ families

Although some providers have lived apart from their families, it is recommended that providers continue to stay at home with their families [33-35]. This preserves everyone’s mental health. Providers should carefully follow standard precautions including utilization of appropriate PPE at work and washing hands as indicated. On arriving home they may change clothes. They should avoid sharing utensils and items of personal use. They should maintain social distance from their family members as necessary, especially if the family members are in the high-risk category.

Providers at risk

Regardless of the degree of symptoms or acuity of illness, substantial environmental contamination is present in air samples and surface samples from the room where a COVID-19 patient is cared for [36]. Infectious aerosol is present even in the absence of procedures known to generate aerosol. It is known that asymptomatic and pre-symptomatic carriers can transmit SARS-CoV-2 virus [37]. Nebulization and noninvasive positive pressure ventilation also generate large amounts of aerosol. CDC has presented the data on the adverse effects of COVID-19 [38].

Providers sensitive to risk

Even though not necessarily at increased risk, some providers want to protect themselves from COVID-19, perhaps more so than their colleagues. Pregnant providers do not want any health issues in the mother or infant. Some providers are sole breadwinners and heads of household with small children or other dependents [5]. Some providers may have psychological aversions.

Risk with inadequate personal protective equipment

Although providers who themselves are in high-risk categories are safe when utilizing appropriate PPE, in its absence they are at substantial risk. CDC recommends that in the absence of appropriate PPE, “exclude healthcare personnel at higher risk for severe illness from COVID-19 from contact with known or suspected COVID-19 patients [39].” Providing care without appropriate PPE should not be a condition of employment for any provider, especially for the ones in high-risk category. The argument that individuals who chose careers in healthcare accepted its potential risks does not apply. 

Providing care despite inadequate personal protective equipment​​​​​​​

Many providers work in settings where they consider the PPE to be inadequate. One approach to providing care in this setting is to incentivize call and make it voluntary. Providers who agree to provide call coverage also agree to care for COVID-19 patients. Those who forego call are not required to care for patients who have tested positive for SARS-CoV-2 and for persons under investigation. In addition to monetary incentives, the on-call person’s responsibilities for caring for patients not known to have COVID-19 may be substantially reduced.

If possible, COVID-19 patients should be cared for in negative pressure rooms with high air exchange rates. The air should also be filtered adequately. Alternatively, rooms with sunlight and natural air flow through open windows may be utilized. In Germany and other locations, many patients are cared for at home with remote monitoring and regular visits from providers.

## Conclusions

Utilizing appropriate PPE is the key to safely providing care for COVID-19 patients. There is a worldwide shortage of PPE. To alleviate it, CDC is allowing extended use, reuse, and reprocessing of N95 masks. Providers are at increased risk and thousands of providers in the United States have been infected with COVID-19. This is in part due to inadequate utilization of PPE. Risk of infection increases with age and presence of comorbidities. African-Americans and Latinos are at increased risk. It is recommended that providers do not stay away from their families but instead follow all precautions. Providers should not be required to provide care in the absence of adequate PPE. This is especially true of providers in high-risk categories.
